# Low-dose dexmedetomidine as a perineural adjuvant for postoperative analgesia: a randomized controlled trial

**DOI:** 10.1186/s12871-022-01791-6

**Published:** 2022-08-05

**Authors:** Wei Liu, Jingwen Guo, Jun Zheng, Bin Zheng, Xiangcai Ruan, Addition of Dexmedetomidine to Ropivacaine-Induced supraclavicular Block (ADRIB) investigator

**Affiliations:** 1 Department of Anesthesiology, School of Medicine, the Second Affiliated Hospital, South China University of Technology, Guangzhou, 510180 People’s Republic of China; 2 Department of Anesthesiology and Pain Medicine, The Sixth Affiliated Hospital, Sun Yat-Sen University, Guangzhou, 510665 China

**Keywords:** Dexmedetomidine, Nerve block, Postoperative pain

## Abstract

**Purpose:**

Dexmedetomidine has been proposed as an additive to local anesthetics to prolong peripheral nerve block duration; however, perineural dexmedetomidine has been associated with an increased risk of bradycardia and hypotension This randomized controlled study investigated the effects of low-dose dexmedetomidine as a perineural adjuvant for postoperative analgesia.

**Methods:**

Fifty-five patients who had undergone elective upper extremity surgery were randomized to receive an ultrasound-guided supraclavicular brachial plexus block with 20 mL 0.5% ropivacaine with or without 30 μg dexmedetomidine. The primary outcome was the duration of analgesia. Secondary outcomes included the onset time and duration of the motor and sensory blocks, incidence of hypotension and bradycardia, total postoperative analgesics, and safety assessment during the 24 h after surgery.

**Results:**

Dexmedetomidine significantly prolonged the duration of analgesia (887 ± 92 min *vs* 661 ± 83 min, *P* < 0.0001). The onset time and the duration of motor and sensory block were significantly different between the groups (all *P* < 0.001). No episodes of hypotension or bradycardia were detected in the dexmedetomidine group. The total postoperative analgesic use and side effect profiles in the first 24 h postoperative period were similar for both groups.

**Conclusions:**

Low-dose dexmedetomidine (30 μg) as a perineural adjuvant significantly prolonged the analgesic duration of a brachial plexus block without inducing hemodynamic instability.

**Trial registration:**

This trial was registered at ClinicalTrial.gov (***NCT02630290***).

## Introduction

The supraclavicular brachial plexus block provides optimal analgesia in patients undergoing upper extremity surgery [1]. However, the duration of analgesia of single-shot nerve blocks is not sufficient for avoidance of moderate to severe postoperative pain. A technically simple strategy for prolonging the duration of single-shot blocks is the use of a perineural adjunct like dexmedetomidine [2]. This alpha-2 adrenoceptor agonist has been shown to improve the quality and duration of brachial plexus blocks [1, 3], and well-designed preclinical studies have also shown that the addition of supraclinical doses of dexmedetomidine (20–40 μg/kg) to local anesthetics is well tolerated without any signs of neurotoxicity [4, 5]. The off-label use of dexmedetomidine as a perineural adjuvant for postoperative analgesia has therefore been a subject of increasing interest, especially within the context of multi-modal analgesia [6–9].

However, perineural dexmedetomidine has adverse side effects, such as bradycardia, hypotension, and sedation, and these are likely dose-dependent [3, 4]. Marhofer et al. [10] recommended a dose of approximately 100 μg dexmedetomidine for perineural administration to balance between the optimization of block characteristics and suppression of the common side effects of dexmedetomidine, such as bradycardia and sedation. Their volunteer studies showed a significant dose-dependent increase in the duration of sensory block as well as a significant dose-dependent side-effects following the use of dexmedetomidine as an adjuvant to ropivacaine [10, 11]. Unfortunately, a study evaluating a perineural dose of 100 μg reported a case of minor nerve damage that persisted for 5 months and a frequent occurrence of episodes of bradycardia and mild to moderate sedation [12]. Previously, a meta-analysis in the setting of brachial plexus block has investigated the clinical effects of perineural dexmedetomidine and suggests an optimal dose of 50–60 μg for the balance [13]. However, subsequent meta-analyses [2, 14, 15], failed to demonstrate a dose-related difference for the clinical effects of perineural dexmedetomidine, partially due to a lack of evidence.

To date, only few studies have focused on the effect of low-dose dexmedetomidine as local Anesthetic adjuncts for peripheral nerve block and much less studies assessed this effect in brachial plexus block. A trial in patients undergoing arthroscopic shoulder surgery under an interscalene brachial plexus block reported that a much smaller dose of perineural dexmedetomidine decreased both postoperative pain scores and rescue analgesic requirements [16]. This was the only study to show that a dose as low as 10 μg was sufficient to achieve a considerable improvement in postoperative analgesia while avoiding the adverse effects associated with systemic dexmedetomidine, although this finding might have resulted from differences in outcome measurements [16]. Another trial [17] adding 30 μg dexmedetomidine to bupivacaine-induced supraclavicular brachial plexus block reported that dexmedetomidine increased the mean duration of analgesia by 2.8 times when compared with placebo. The trial also noted several cases of bradycardia and hypotension in the dexmedetomidine group; however, no detailed hemodynamic information was provided [17]. Nevertheless, these studies suggest that smaller doses of dexmedetomidine can increase the analgesic duration of brachial plexus block, and their results need to be confirmed.

We designed a randomized-controlled study to evaluate the effects of perineural administration of 30 μg dexmedetomidine on the analgesic duration of a brachial plexus block in patients undergoing upper extremity surgery. Our secondary outcomes included the onset time and duration of the motor and sensory blocks, the incidence of hypotension and bradycardia, the total postoperative analgesic use, and safety assessments.

## Materials and methods

The ADRIB (Addition of Dexmedetomidine to Ropivacaine-induced supraclavicular Block) study was conducted in accordance with the Consolidated Standards of Reporting Trials (CONSORT) statement and the Declaration of Helsinki. Our study protocol was approved (28/09/2015) by the Guangzhou First People’s Hospital Research Ethics Board and was registered on clincaltrials.gov (NCT02630290; principal investigator: XR, date of registration: 15/12/2015). The protocol is available by request from the corresponding author. Written informed consent was obtained prior to study enrollment. Participants were recruited from Guangzhou First People’s Hospital, a tertiary teaching hospital affiliated with South China University of Technology. Adult patients aged 18–60 years with an American Society of Anesthesiologists (ASA) physical status 1 or 2 who were scheduled for elective forearm and hand orthopedic surgery were recruited. Written informed consent was obtained from each patient. Exclusion criteria were: taking antihypertensive drugs such as methyldopa, clonidine, and other α2 receptor agonists; peripheral neuropathy; coagulopathies; liver and kidney dysfunction; or history of allergy or hypersensitive reaction to any of the study medications.

Computer generated block randomization in blocks of 10 subjects in a 1:1 ratio was used to randomly allocate the patients to an ultrasound-guided supraclavicular brachial plexus block with 20 mL 0.5% ropivacaine with or without 30 μg dexmedetomidine. The group allocation was concealed in sequentially numbered and sealed opaque envelopes. A nurse who was not involved in any other sections of the study obtained the envelopes before administration of the nerve block and prepared the treatment solutions by diluting 100 mg of ropivacaine (AstraZeneca AB, Sodertalje, Sweden) with or without 30 μg of dexmedetomidine (Hengrui Med, Jiangsu, China) in 0.9% sodium chloride in a final volume of 20 mL.

All the patients received 1.5 mg midazolam i.v. after peripheral intravenous access was secured. ECG, noninvasive arterial blood pressure, and pulse oximetry were monitored. An ultrasound-guided supraclavicular nerve block was performed according to a previously described technique [18]. With the patient in the supine position and after skin disinfection, a linear ultrasound probe was positioned in the supraclavicular fossa inferomedially to identify the subclavian artery, the brachial plexus, and the pleura. The skin and the subcutaneous tissue were infiltrated with 3 mL 2% lidocaine, and a 20-gauge needle (0.90 × 90 mm) was inserted from lateral to medial using an in-plane technique until the tip of the needle was located deep between the subclavian artery and the first rib. A 5 mL volume of the treatment solution was injected, and the needle was redirected to the intermediate and superficial levels of the brachial plexus for a maximum spread of the rest treatment solution. All blocks were performed by a single trained investigator. The patients and the anesthesiologist performing the block were blinded to the study treatments.

Each patient’s heart rate (HR), noninvasive blood pressure, and oxygen saturation were recorded every 5 min until the end of the surgery and then again every 30 min up to 24 h postoperatively. The incidence of bradycardia (HR < 50 beats/min), hypotension (30% or more decrease in mean arterial pressure from baseline values), or hypoxemia (SpO2 < 90%) was noted and managed by atropine, vasopressor, or oxygen mask, respectively. We assessed the sensory block using a 3-point pinprick scale (0 – 2, 0 = normal sensation, 1 = decreased pain sensation, 2 = loss of pain sensation) in the median, ulnar, radial, and musculocutaneous nerve locations every 5 min until 30 min after administration of the block. We evaluated the motor block in these 4 nerves using a thumb and second finger pinch, thumb and fifth finger pinch, finger abduction, and flexion of the elbow, respectively. We defined the onset time of the sensory block as the time from the local anesthetic injection until the loss of pain sensation in all 4 nerve territories and the onset time of the motor block as the time from the local anesthetic injection until the absence of movements in the hand and forearm. If the patient experienced pain in the surgical area, local anesthetic (2% lidocaine) infiltration was added by the surgeon. The need for supplementation of the block was at the discretion of the anesthesiologists.

Upon completion of the surgery, the patients were returned to surgical wards and monitored for 24 h by an investigator blinded to the group allocation. HR and blood pressure were recorded every hour for 6 h, then every 2 h until 12 h, and once more at 24 h postoperatively. Postoperative pain was assessed using a visual analog scale (VAS, 0–100, 0 = no pain, 100 = maximum imaginable pain) at the same time intervals. Tramadol (50 mg) was administered when the postoperative VAS > 3, and was titrated up by 50 mg increments as needed for pain every 4 to 6 h, with the maximum total dose of 250 mg per day. The duration of analgesia was defined as time since local anesthetic injection until the report of postoperative pain at the surgical site, and the total analgesic requirement in first postoperative 24 h was also recorded. Incidences of postoperative nausea and vomiting (PONV) were managed with ondansetron. Any patient's self-reported abnormal sensation in the hand and/or forearm was recorded.

### Statistical analysis

The primary outcome measure of this study was the duration of analgesia. The sample size calculation was based on a pilot study noting a mean value of 660 min and SD of 200 min for the duration of analgesia using a 2-sample t test with a 0.05 two-sided significance level and a power of 0.8. A 25% difference in the duration of analgesia was regarded as clinically relevant. We calculated that a minimum sample size of 27 patients was required in each group. This was increased to 30 patients to compensate for possible dropouts.

The independent 2-sample t test was used to compare normally distributed data and the Mann–Whitney U test was used to compare categorical or skewed data between the groups. Repeated-measures analysis of variance, followed by post hoc analysis with Bonferroni correction, was used to compare HR and blood pressure. The chi-square or Fisher's exact tests were used to compare the incidence of adverse effects. A value of *P* < 0.05 was regarded as statistically significant.

## Results

**Fig. 1:**
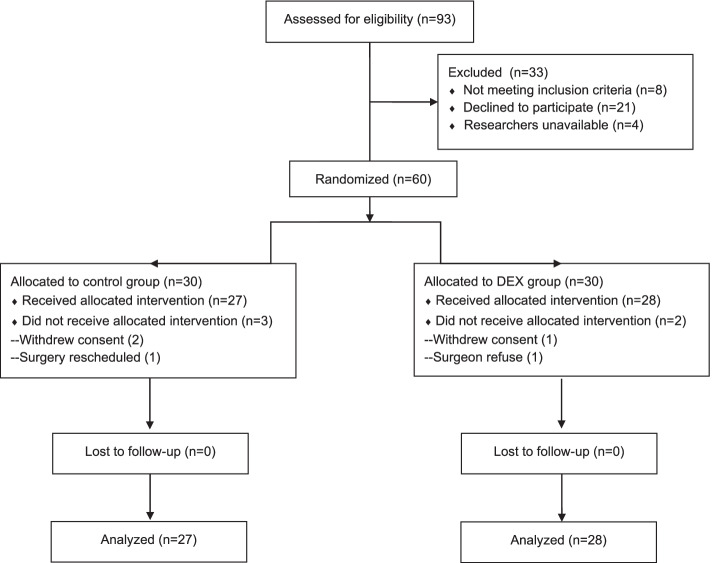
CONSORT flow diagram showing number of patients at each phase of the study

Sixty patients provided written informed consent and were randomly assigned to either the control group or the dexmedetomidine group. After allocation, 55 patients (27 in the control group and 28 in the dexmedetomidine group) completed follow-up and were included in the intention-to-treat analysis (Fig. 1). The groups were comparable with respect to demographic and surgical characteristics (Table 1).

**Table 1:** Demographic and surgical characteristics. Values are mean ± SD or number

	Control group (*n* = 27)	Dexmedetomidine group (*n* = 28)
Age (years)	40 ± 12	37 ± 11
Sex (M/F), *n*	15/12	13/15
Weight (kg)	61 ± 10	65 ± 15
ASA I/II ( *n*)	22/5	22/6
Duration of surgery (min)	82 ± 46	65 ± 23

**Fig. 2:**
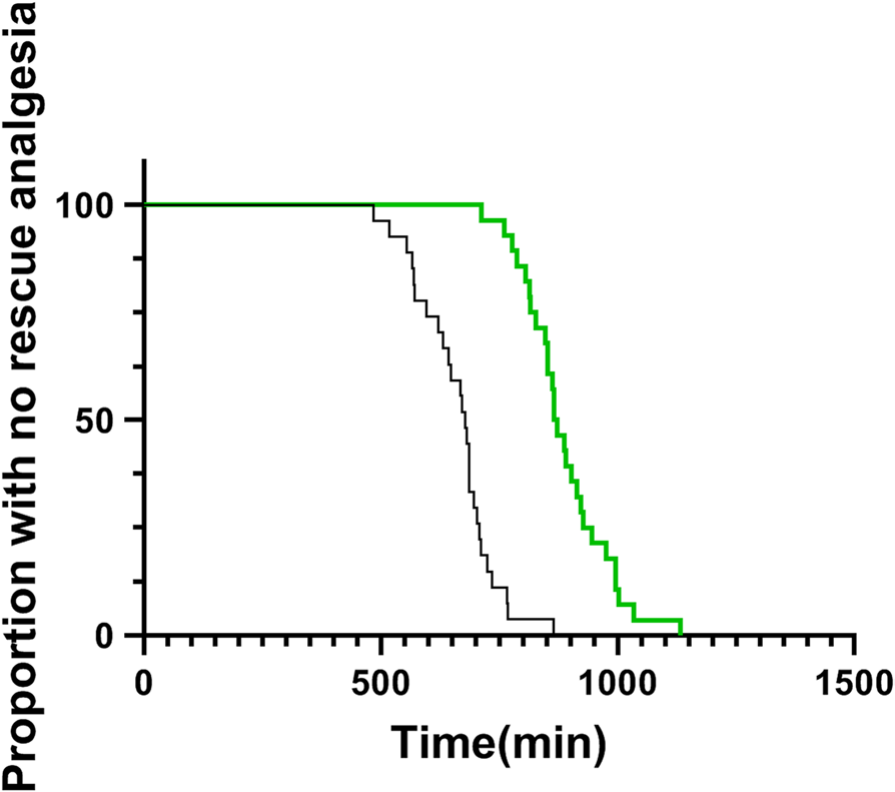
Kaplan–Meier survival curves of time to first use of rescue medication in patients receiving control (  ) or dexmedetomidine (  )

**Fig. 3:**
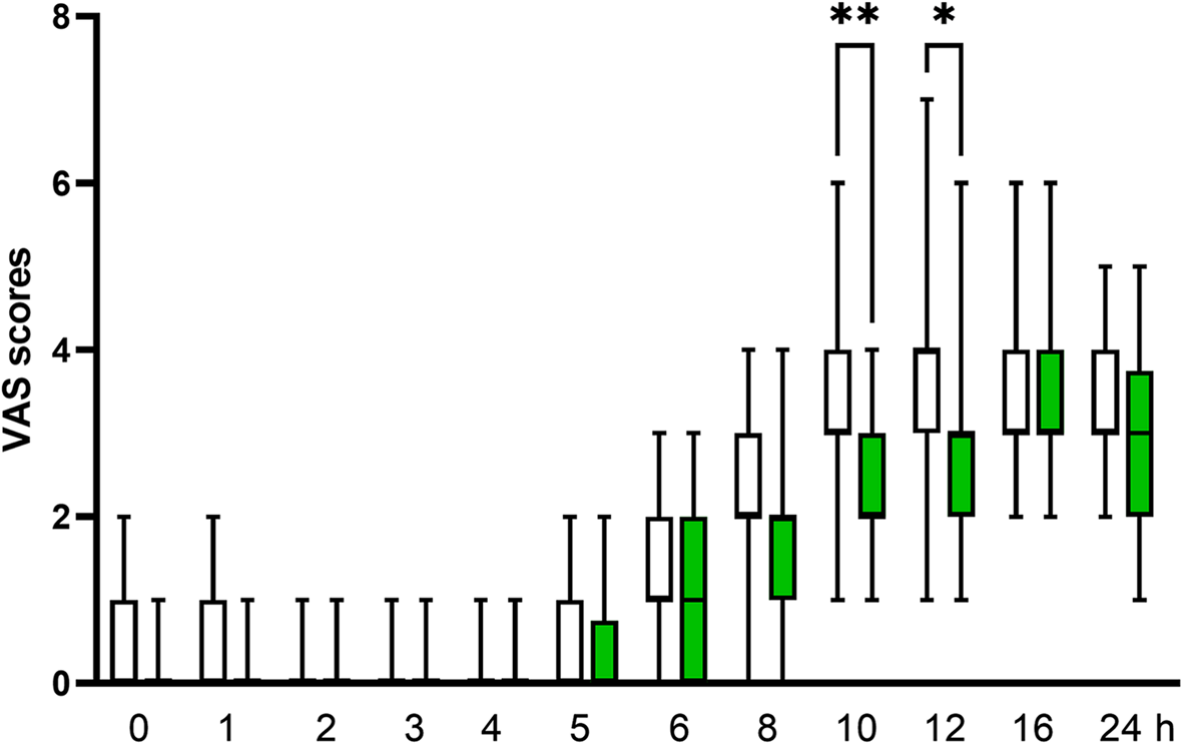
Postoperative pain scores in patients receiving control (  ) or dexmedetomidine (  ). Boxes indicate median with 25th and 75th percentiles (interquartile range) and whisker caps indicate minimum to maximum values. *P < 0.05, **P < 0.01. VAS, visual analog scale

As shown in Table 2, the block performance time and the number of block attempts were comparable in both groups. None of the patients in either group required local anesthetic infiltration or block supplementation during surgery. The duration of analgesia was significantly longer in the dexmedetomidine group than in the control group (*P* < 0.0001, Table 2 and Fig. 2). The addition of dexmedetomidine to ropivacaine also decreased the onset time and prolonged the duration of the sensory and motor blocks compared with the control (all *P* < 0.0001). Postoperative VAS scores were similar for both groups at the same time assessment points, except at 10 and 12 h postoperatively when the VAS scores were lower in the dexmedetomidine group than in the control group (Fig. 3). However, total requirement of tramadol and number of patients requiring analgesics during the 24 h postoperative period were comparable between group (*P* > 0.05).

**Table 2:** Nerve block characteristics and incidence of hemodynamic side-effects. Values are mean ± SD or number (proportion)

	Control group (*n* = 27)	Dexmedetomidine group (*n* = 28)	*P* value
Block performance time (min)	4 ± 1	4 ± 1	0.939
Onset of sensory block (min)	21 ± 4	16 ± 3	0.000
Onset of motor block (min)	25 ± 3	19 ± 4	0.000
Duration of sensory block (min)	571 ± 90	783 ± 108	0.000
Duration of motor block (min)	612 ± 90	865 ± 104	0.000
Duration of analgesia (min)	661 ± 83	887 ± 92	0.000
Cumulative tramadol consumption during 24 h after surgery (mg)	33 ± 44	20 ± 37	0.215
Number of patients requiring tramadol during 24 h after surgery	12 (44%)	7 (25%)	0.162
Incidence of PONV	3 (11%)	6 (21%)	0.469
Incidence of hypotension	1 (4%)	0	0.491

The pre-operative and intra-operative changes in systolic arterial blood pressure and heart rate were comparable between the groups. No episodes of bradycardia were observed, and a single patient in the control group experienced transient hypotension which required no intervention. Although the dexmedetomidine group had more patients experiencing PONV than the control group during the 24 h postoperative period, the difference was no statistical significance (6/27 vs 3/28, *P* = 0.469, Table 2). No episodes of respiratory depression, hypoxia, or block-related side-effects were encountered during the study period.

## Discussion

This study evaluated the use of a perineural adjuvant to local anesthetics within the context of multi-modal analgesia. The findings showed that low-dose dexmedetomidine significantly prolonged the duration of the nerve block, as well as the duration of analgesia. Furthermore, the perineural adjuvant did not induce hemodynamic instability, and no patient developed any symptoms of neurotoxicity.

Dexmedetomidine appears to be a beneficial adjuvant to the long-acting local anesthetics used in nerve blocks. The findings presented here suggest that a low dosage perineural administration should also be considered to minimize the risk of the common adverse side-effects of dexmedetomidine. Our data are generally consistent with the previous meta-analysis [7] showing that a 50–60 μg dose of dexmedetomidine added to the long-acting anesthetics used to induce a brachial plexus block minimizes the risk of hemodynamic events, such as hypotension and bradycardia. The low-dose strategy proposed in the current study has been employed in one previous volunteer study, and showed that 20 μg dexmedetomidine added to the 0.75% ropivacaine used to induce an ulnar nerve block did not have any hemodynamic side effects [10]. However, extrapolation of volunteer results to the clinical setting is always difficult [19]. We found an approximately 40% extension of the duration of sensory block with the adjunct of 30 μg dexmedetomidine to the ropivacaine-induced brachial plexus block in our patients, whereas the volunteer study reported a 60% extension with the adjunct of a 20 μg dose to the ropivacaine-induced ulnar nerve block [10]. By contrast, Abdulatif et al. [20] found no increase in the duration of postoperative analgesia with the use of 25 μg dexmedetomidine as an adjuvant to a femoral nerve block for arthroscopic knee surgery, whereas the use of 50 μg dexmedetomidine extended the time to first request for postoperative analgesia by 102%. These discrepancies could be attributed to the size of the nerve targeted, the type of local anesthetic used, the patient population selected, and the general anesthesia combination.

The local neurotoxicity of perineural administration of dexmedetomidine has been well investigated in experimental studies [21, 22] and shows that the medicine regimen up to a supraclinical dose of 40 μg/kg has no effect on nerve axons or myelin [5]. A recent meta-analysis evaluating perineural dexmedetomidine in the setting of brachial plexus nerve blocks also confirmed the safety profile of perineural dexmedetomidine [7]. However, sporadic transient nerve damage in healthy volunteer studies has been associated with the use of perineural high-dose dexmedetomidine and has raised safety concerns. For example, Keplinger et al. [11] reported that two volunteers receiving 150 μg dexmedetomidine (perineural concentration of 33 μg/mL) experienced paresthesia that lasted for 3 days after an ulnar nerve block. They speculated that the high dose of perineural dexmedetomidine may be responsible for the prolonged paresthesia. Similarly, Andersen et al. [12] found that another volunteer receiving 100 μg dexmedetomidine (perineural concentration of 5 μg/mL) experienced a paresthesia 5 months in duration after a saphenous nerve block. Several reasons could explain the lack of neurologic deficit in the patients receiving perineural dexmedetomidine observed in the present study. One was that we avoided high doses and cautiously diluted the low dose to a 20 mL solution, yielding a relatively lower perineural concentration of 1.5 μg/mL dexmedetomidine in the block. Some uncertainty remains regarding a possible concentration dependence of neurologic events; therefore, this requires further confirmation. A second reason was our use of a strict ultrasound-guided brachial plexus injection technique, which can reduce the risk of inadvertent needle trauma and intraneural injection.

How a local anesthetic adjunct like dexmedetomidine prolongs the duration of a nerve block remains unclear. Both animal [23] and human volunteer [12] data indicate that dexmedetomidine increases the duration of analgesia through direct perineural mechanisms. One speculation is that peripheral perineural dexmedetomidine directly blocks hyperpolarization-activated cation currents and resets the hyperpolarized nerves back to a resting membrane potential [23]. Interestingly, a recent clinical study with a triple-group design reported that systemic dexmedetomidine was not inferior to perineural dexmedetomidine in prolonging the analgesic duration of an interscalene brachial plexus block for ambulatory shoulder surgery and in decreasing the pain and opioid consumption up to 8 h postoperatively [24]. The finding of a systemic route that is equivalent to perineural administration may be a result of analgesic effects of dexmedetomidine following systemic infusion rather than peripheral effects of the nerve block. However, other work by the same author published in last year indicated that intravenous dexmedetomidine did not affect peripheral nerve block characteristics [2]. Nevertheless, A systematic review of dexmedetomidine as a local Anesthetic adjuvant published this year provides an excellent comprehensive summary of the published literature to date on this topic. This review suggests intravenous dexmedetomidine as a peripheral nerve block adjunct was inferior to perineural dexmedetomidine [14]. While the present study supports the concept that the analgesic effects of low-dose dexmedetomidine are primarily peripheral, further investigations are needed to confirm our results and to identify the exact mechanism of action.

Our study was not sufficiently powered to detect the differences in common adverse effects of dexmedetomidine between groups. Nevertheless, the adverse effects, such as bradycardia, hypotension, and sedation, following the administration of dexmedetomidine are very likely dose-dependent [4, 20]. The lack of a difference in the incidence of adverse events may reflect the designated dosage that we used. The trail investigated supraclavicular brachial plexus block for upper extremity operations, which decrease the external validity and generalizability of our findings.

In conclusion, the present study shows that low-dose dexmedetomidine used as a perineural adjunct prolongs the duration of postoperative analgesia without producing significant hemodynamic instability in patients receiving an ultrasound-guided supraclavicular nerve block.

## Data Availability

Data are available on reasonable request to the correspondent author.
